# Study on Regional Strata Movement during Deep Mining of Erdos Coal Field and Its Control

**DOI:** 10.3390/ijerph192214902

**Published:** 2022-11-12

**Authors:** Guojian Zhang, Zhiyang Wang, Guangli Guo, Wei Wei, Fugang Wang, Leilei Zhong, Yaqiang Gong

**Affiliations:** 1 School of Surveying and Geo-Informatics, Shandong Jianzhu University, Jinan 250101, China; 2 Post-Doctoral Workstation of Technology Research Institute, Shandong Energy Group Co., Ltd., Jinan 250101, China; 3 School of Environmental Science and Spatial Informatics, China University of Mining and Technology, Xuzhou 221116, China; 4 Yankuang Energy (Erdos) Co., Ltd., Kangbashi District, Ordos 017010, China; 5 Yingpanhao Coal Co., Ltd., Ordos 017300, China

**Keywords:** surface subsidence control, strata movement control, local filling, main key strata

## Abstract

Disasters such as rock bursts and mine earthquakes became increasingly serious with the increase in mining depth in Erdos Coal Field and became serious problems that restrict high-strength continuous mining of coal mines. In this study, strata movement and energy polling distribution of ultrathick weak-bonding sandstone layers were controlled by the local filling–caving multi-faces coordinated mining technique, which was based on the analysis of subsidence and overlying structural characteristics in the Yingpanhao mining area. Moreover, the influencing factors and the control effect laws were investigated. Surface subsidence and energy polling distribution control effects of different mining modes were compared, which confirmed the superiority of local filling based on the main key stratum. According to the results, the maximum surface subsidence velocity of the first mining face was 1.24 mm/d, which indicates the presence of a logistic functional relationship between the mining degree and subsidence factors. When the mining degree was close to full mining, the practical surface subsidence was smaller than the corresponding logistic functional value. The largest influencing factor for the strata movement control effect of partial filling mining based on the main key stratum was the width of the caving face, followed by the filling ratio, section pillar width, and width of the filling face, successively. With respect to the influencing degree on the energy polling distribution of partial filling mining based on the main key stratum, the order followed as section pillar width > filling ratio > caving working face > width of backfilling working face. Additionally, the comparative analysis from the perspectives of control effect, resource utilization, and cost-effectiveness demonstrated that partial filling mining based on the main key stratum was one of the techniques with high cost-effectiveness in controlling strata movement and relieving rock bursts, mining earthquakes, and subsidence disasters.

## 1. Introduction

The deep mining areas of Erdos Coal Field are ideal for high-strength large-scale mining of deep coal resources because of their rich coal reserves, simple geologic structures, extensive territory, and small population. At the same time, high-strength large-scale mining of coal resources in the deep mining areas of Erdos Coal Field is facing various problems, such as rock bursts, frequent occurrences of mining earthquakes, lowering of groundwater levels, and serious surface salinization. Therefore, it is crucial to study the regional strata movement control during the deep mining of Erdos Coal Field. At present, common strata movement control techniques mainly include techniques centred on the filling body and coal–rock pillars.

Research on strata movement control techniques centred on coal–rock pillars mainly focuses on the stability of coal pillars and coal pillar-overlying strata collaborative deformation. For example, considering the coal pillar loads, the effective region theory, pressure arch theory, and two-region constraint theory could completely interpret the stress concentration in coal pillars and overlying strata under different mining conditions [1]. Considering the strength of coal pillars, A.H. Wilson [2,3,4,5,6] and Wu Lixin et al. [7] discussed the theory of strength inequality in core areas, simplified the formula of ultimate strength, and proposed the theory of the “platform loading method”. B.A. Poulsen [8,9,10], RK Wattimena [11], MA Idris [12], AHSG and Munro [13,14,15], E Ghasemi [16], Mehdi Najafiji [17], and Lu Paul [18] et al. investigated the influencing factors and evaluation methods for the stability of coal pillars. Various strata control theories, such as coal pillar compression and the press-in hypothesis [19], rock beam hypothesis [20], and supporting plate theory [21,22,23], were proposed for coal pillar-overlying strata structural collaborative deformation. Zhang Ming et al. analyzed the stability of coal pillars, as well as the collaborative deformation mechanism of ultrathick strata and coal pillars that remained after mining the ultrathick conglomerate working face in deep wells. The stress–strain between coordinated deformation of the ultrathick strata–coal pillar system was presented. The study’s reasonability was preliminarily validated using a comparative analysis between research results and measurement data [24]. Jiang Fuxing carried out a case study on deep strip mining (120 m width). By constructing a separation layer mechanical model of the key stratum, he analyzed the relationship between the ultimate span of the key stratum and face scale, as well as the relationship between the deflection of the key stratum and compression of the coal pillar below the key stratum. The study demonstrated that the separation layer scale of the key stratum was positively related to the ultimate span of the key stratum but negatively related to the deflection of the key stratum [25].

Research on strata movement control techniques was centred on the filling body, mainly on compressive deformation of the filling body and composite support, as well as strata movement during filling mining and relevant control. For compressive deformation of gangue, Guo Guangli [26] and Wang Lei [27] et al. proposed the concepts of effective filling thickness and equivalent mining thickness of gangue filling mining by combining the compressive deformation of the gangue filling body and the overlying strata movement and disclosed the subsidence reduction mechanism of gangue filling mining. By focusing on the size grading of gangue, Zhang Jixiong [28] and Jufeng [29] et al. discussed the macroscopic mechanical properties and microscopic evolutionary characteristics. It disclosed the influencing mechanism of load-bearing compressive deformation of gangue filling materials. Liu Ding investigated the influence of groundwater infiltration on the creep mechanical characteristics of the gangue-cemented filling body and constructed a fractional order constitutive model that could describe the whole creep process [30]. Xin Hengqi designed a confined compression test and determined the load-bearing mechanical properties of broken gangue under the infiltration state [31]. Qian Ziwei carried out confining pressure and creep observation experiments of broken gangues under different moisture contents while revealing the infiltration-saturated yielding mechanism of gangue [32]. Ma Zhanguo conducted an experimental study on the mechanical properties of saturated gangues to understand the relationships between stress and strain [33]. Moreover, they determined the porosity and cracking–swelling factor, as well as the compression characteristics of saturated gangues.

Considering the collaborative loading of the deep filling body–coal pillar composite carrier, Wang Fangtian investigated the influences of filling ratio and strength on filling body–coal pillar stress distribution and fracture development laws of the overlying strata in the ultrahigh filling working face by using the discrete element PFC numerical simulation software. They disclosed the ultrahigh water-filling body–coal pillar collaborative bearing mechanism by combining theoretical analysis. Moreover, when the filling ratio of ultrahigh water filling materials was higher than 90% and the water–cement ratio was lower than 95%, the filling body–coal pillar could achieve the optimal collaborative bearing effect and could effectively control the overlying strata movement [34]. Based on the disperse continuous medium coupling principle, Guang Guangli investigated the stress loading and failure characteristics of the composite carrier during deep stripe filling mining. Based on theoretical analysis of the evolutionary laws and characterization parameters of stress concentration in the filling body and coal pillars in the filling body–coal pillar collaborative bearing process, he also analyzed the influences of filling body and filling workface scale on the filling body–coal pillar collaborative bearing effect. The results provide a reference for the design of a deep stripe-filling mining scheme [35].

Research concerning filling mining-induced strata movement and control mainly focuses on full filling in the goaf and partial filling mining based on the key stratum structure. Studies about strata movement laws and control during partial filling mining based on the key stratum structure mainly focus on local filling in the goaf, or filling practices in the caving zone or overlying strata separation zone formed after the collapse of the direct roof, and realized three aspects of subsidence reduction through joint control over the filling body, separation coal pillars, and key stratum structures of overlying strata [36]. For instance, Guo Guangli [37], Ge Haibin [38], and Zhou Xuwen [39] believed that the grouting filling in the caving zone formed after the collapse of the direct roof could form new bearing structures to withstand the overlying strata loads. The strip-filling mining proposed by Xu Jialin [40], the “mining-filling-leaving” combined coordinated mining technique proposed by Dai Huayang [41] and Guo Junting [42] et al., and the green coordinated mining technique of the “strip mining roadway filling method” proposed by Bai Erhu [43] and Guo Wenbing [44] are all used for control surface subsidence with the spatial structure of the “filling stripe-overlying strata-main key stratum” formed by the strip filling face and overlying structure. Zhu Weibing [45] and Bai Jinwen [46] et al. proposed a technical method of filling strata control beside the key pillar and elaborated the basic control theory of strata movement.

Considering studies on strip mining and partial filling mining, much research was conducted to construct collaborative mechanical models of coal pillars, composite carriers and key layer structures of overlying strata. It could provide references to study regional strata movement during deep mining of Erdos Coal Field. Moreover, existing research results demonstrate that ultra-thick sandstone in overlying strata in the deep mining areas of Erdos Coal Field had a strong control effect. These strata movement and energy polling distribution characteristics are some of the major sources of rock bursts and frequent mining earthquakes during large-scale continuous mining.

Hence, a local filling–caving multi-faces coordinated mining method was proposed to control regional mining-induced strata movement in deep areas. The goal was to determine the coordinated development between high-efficiency safety exploitation of coal resources in the deep regions of Erdos Coal Field and surface ecological environmental protection. Additionally, the intention was to decide the influence of ultra-thick sandstone in deep overlying strata with high considerations for mining efficiency and filling cost. Meanwhile, influencing factors and response laws were discussed.

## 2. Analysis of Subsidence and Overlying Strata Structural Speciality in Mining Areas

Subsidence monitoring and hole detection in mining areas are important means to understand strata movement principles in the region. In the following text, the strata mining deformation laws and overlying structural characteristics in deep mining areas in the east (Jining Coal Field and Yanzhou Coal Field) and west (Erdos Coal Field) were compared through monitoring data. The results provided reference data for designing regional strata movement control during deep mining of ultrathick weak bonding overlying strata in Erdos Coal Field.

### 2.1. Comparative Analysis of Strata Movement Characteristics in the East and West Deep Mining Areas

**Figure 1:**
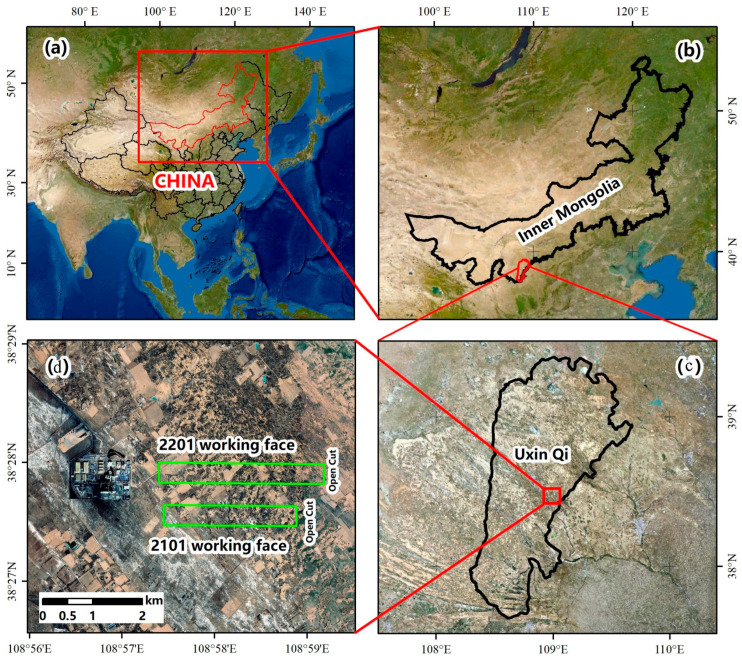
Location of the study area. (**a**) China. (**b**) Inner Mongolia. (**c**) Uxin Qi. (**d**) Working face.

In this study, Yingpanhao Coal Mine was chosen as the major research object. In Yingpanhao coal mine, the caving mining and full-seam mining method are used to manage the roof now. The coal cutter model is MG1100/3050-WD, the hydraulic support type is ZY20000/33.5/68D, and the working face end support model is ZYD17000/24/45D. Its geographical position and mining progress are shown in Figure 1. The Yingpanhao Coal Mine is exploring the 2-2 coal seam, Section 3, Yan’an Formation, while the 2201 working face in Zone 22 and the 2101 working face in Zone 21 were finished. The 2201 and 2101 working faces were about 300 m long, and their advancing lengths were 1806 m and 1983 m, respectively. The coal seams on working faces were horizontal and about 6 m thick. The distance between the 2201 and 2101 working faces was about 300 m.

**Figure 2:**
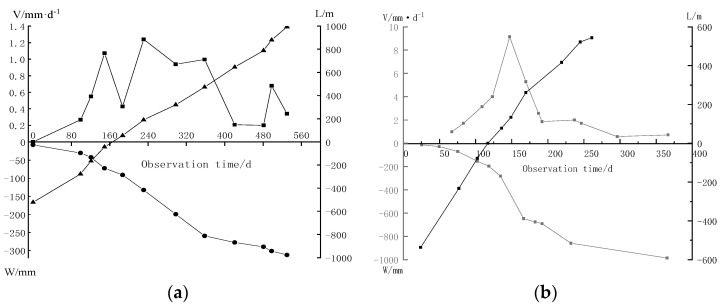
Subsidence velocity of maximum subsidence point of strike line; (**a**) C46 point in the Yingpanhao Coal Mine. (**b**) S30 point in Jining Well 2.

To analyze the special mining-induced movement and deformation laws in the deep mining areas of Erdos Coal Field, the C46 point on the 2201 working face in the Yingpanhao Coal Mine and the S30 point on the 3305 working face in Jining Well #2 (Ji’ning Coalfield, Shandong Province, China) were chosen for comparison. Subsidence and velocity curves at corresponding points were obtained through data processing (Figure 2). V/t represents the subsidence velocity, l/t represents the horizontal distance from the monitoring point to the stope face, and W/t refers to the subsidence value at the monitoring points. Note that the measured data in the paper were obtained by leveling, and the measurement process follows the second class leveling specification.

As shown in Figure 2a, the subsidence velocity at maximum subsidence points under the geological mining conditions was small and the maximum subsidence velocity was only 1.24 mm/d, which was lower than the minimum (1.67 mm/d) in the active subsidence phase. The subsidence velocity increased from 0.55 mm/d to 1.07 mm/d during the observation from 121 d to 231 d, followed by a sharp decline to 0.43 mm/d and then recovering to 1.24 mm/d. After analyzing the overlying strata failure laws, periodic failure of thick sandstone in the Zhiluo Formation during mining activities was found to be the major cause of local surface movement [47].

As shown in Figure 2b, with the advancement of working faces, the subsidence velocity at surface points first increased, followed by a decline at a later stage. The subsidence velocity reached the maximum speed of mining (9.2 mm/d) when the working face advanced over the point by 134 m. After extensive observation, it was found that the movement of this point lasted for 13 months, and the surface subsidence had distinct phases: the initial phase, active phase, and recession phase. In the active phase (subsidence velocity at measuring point >1.67 mm/d), the subsidence volume at S30 could reach 91% of the total subsidence volume. In other words, the subsidence at the surface point mainly occurred in the active movement phase. During the initial phase (subsidence velocity at measuring point <1.67 mm/d), the subsidence volume was found to be only 8% of the total subsidence volume. During the recession phase (subsidence velocity at a measuring point <1.67 mm/d), subsidence volume was found to be only 10% of the total subsidence volume.

Additionally, a genetic algorithm was used to fit the measured data of the observation line subsidence along the surface strike and the probability integral method was inversed to calculate parameters. The results demonstrate that when the 2201 working face advanced by 1634 m, the major influencing angular tangential and deviation of inflection points in the surface movement basin were in accordance with the reference ranges determined in the Guideline for Buildings, Water Body, Railway and Major Roadway Coal Pillar Setting, and Coal Mining (Table 1). However, the subsidence rate was only 47.5% of the reference range lower limit, indicating that the overall movement deformation in the region was relatively small.

**Figure 3:**
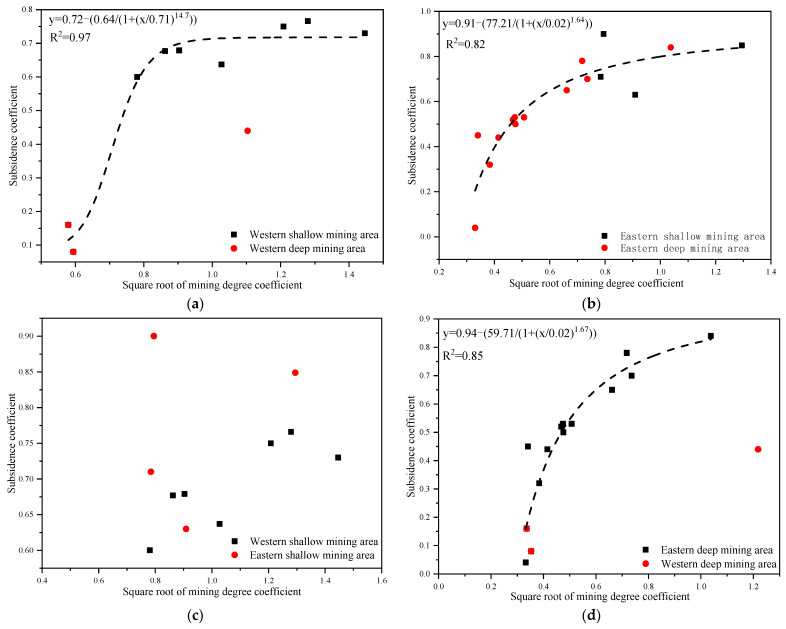
Scatter diagram of subsidence factors in the east and west mining areas; (**a**) scatter diagram of subsidence factors in the west regions. (**b**) Scatter diagram of subsidence factors in the middle and east region. (**c**) Scatter diagram of subsidence factors in the east and west shallow mining areas. (**d**) Scatter diagram of subsidence factors in the east and west deep mining areas.

Surface measured data for the shallow and deep mining areas in the east and west were collected to thoroughly examine the difference in the coal mining-induced surface movement and deformation laws. Moreover, corresponding surface subsidence factors of coal mines were obtained through inversion (Table 2). For an intuitive comparison of the differences in the surface subsidence factors in the east and west mining areas, a scatter diagram of mining degree and subsidence factor was plotted according to the data in Table 2 (Figure 3).

Figure 3a,b demonstrates that the subsidence factor usually presented logistic functional growth with the increase in mining degree in the east and west mining areas. However, when the mining degree approached and reached full mining, the surface subsidence factor in the east deep mining areas was found to be close to that in the shallow regions under the same mining conditions. The mining degree and subsidence factor were consistent with the logistic function. In contrast, the surface subsidence factor in the west deep mining area was far lower than that in the shallow mining areas, while the mining degree and subsidence factor did not comply with the logistic function. As shown in Figure 3c,d, the surface subsidence factors in the east shallow and deep mining areas were generally higher than those in the west shallow and deep mining areas under the same mining conditions. Figure 3d reflected that the subsidence factors of the east and west deep mining areas achieved logistic functional growths with the increase in mining degree. During the early mining of coal seams, the surface subsidence factor of Panyinghao Coal Mine was close to that of Anju Coal Mine. There was magmatic rock invasion into the strata of Anju Coal Mine and the mean thickness reached 124.05 m, belonging to the hard overlying mining area. Therefore, when the surface was in an extremely insufficient mining state, the ultrathick and weak-bonding overlying strata movement during the deep mining of the Yingpanhao Coal Mine was found to have the characteristic of hard overlying rocks. When the mining degree approached and reached full mining, the surface subsidence factor in the west deep mining areas was determined to be smaller under equal mining conditions, while showing harder overlying strata.

### 2.2. Comparative Analysis of Overlying Structural Characteristics in the East and West Deep Mining Areas

For comparative analysis of the overlying strata structural characteristics between the east and west deep mining areas, a case study based on Jining Coal Field and Dongsheng Coal Field was carried out. Some strata structural distribution characteristics of Yingpanhao Coal Mine, Bayan Gaole Coal Mine, and Nalinhe Well #2 in the deep mining areas of Dongsheng Coal Field are listed in Table 3. Some strata structural distribution characteristics of Anju Coal Mine, Tangkou Coal Mine, and Jining #2 Well in the deep mining areas of Jining Coal Field are listed in Table 4.

**Figure 4:**
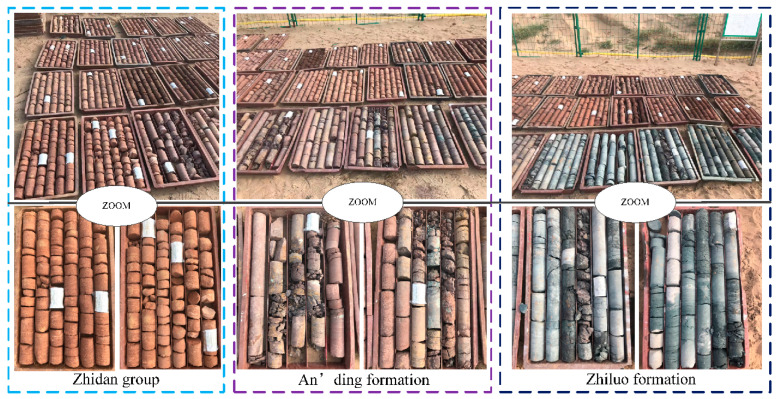
The rock samples from the surface to the boreholes on the coal seam.

According to the borehole and three-dimensional earthquake disclosure, Dongsheng Coal Field was composed of Upper Triassic Yanchang Formation, Lower Jurassic Fuxian Formation, Lower-Middle Jurassic Yan’an Formation, Middle Jurassic Zhiluo Formation, and An’ding Formation. It also consisted of the Lower Cretaceous Zhidan Group, Paleogene and Neogene, and the quaternary system (from old to new) without folds and faults. Some rock samples from the surface to the coal seam borehole are shown in Figure 4. The Jining Coal Field was composed of Middle Carboniferous Benxi Formation, Upper Carboniferous Taiyuan Formation, Lower Permian Shanxi Formation, Lower Shihezi Formation, Upper Permian Upper Shihezi Formation, Upper Jurassic Santai Formation, as well as Palaeogene, Neogene, and quaternary systems from old to new. Among them, the Jurassic system was the major coal-bearing stratum in the Dongsheng Coal Field, while the coal-Permian system was the major coal-bearing stratum in the Jining Coal Field.

To further determine the structures and physical as well as mechanical properties of the main key stratums in the overlying strata, sandstones from the Zhidan Group, Anding Group, Zhiluo Group, and Yan’an Group were selected for component analysis test, mechanical test, scanning electron microscope (SEM) test, and Qinshui test. According to the test results (Table 5), although sandstones in the Zhidan Group were relatively soft, rich porosity was present. Most bonding materials were calcite cementation, accompanied by a few clay minerals. Cement composition was almost consistent with detritus, which would not disintegrate in water. The strata were relatively thick, while fractures and vertical joints were hardly developed. There were hardly any faults or folds. The strata typically had great rigidity while serving as the foundation layers of the overlying rock structure. Please see the detailed mechanical properties and characteristics of the overburden in reference [48].

## 3. Design of the Regional Strata Movement Control Scheme during Local Filling–Caving Multi-Faces Coordinated Mining in Deep Areas

**Figure 5:**
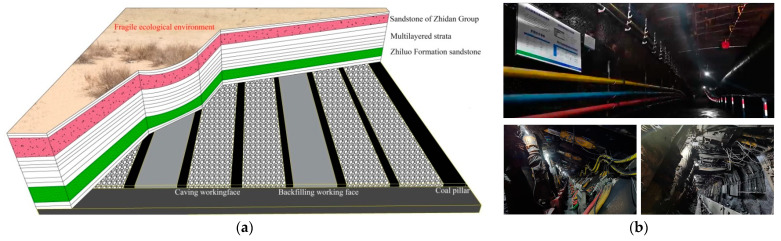
Local filling–caving multi-faces coordinated mining in deep areas; (**a**) schematic design. (**b**) Underground live.

According to the overlying structural characteristics and the physical, as well as the mechanical, properties of weak bonding sandstone in the deep mining areas of Erdos Coal Field, the local filling–caving multi-faces coordinated mining method based on the main key stratums was proposed. It could decrease the intensity of the dynamic appearance of overlying rocks and the degree of surface failure. The mining scheme design is shown in Figure 5.

**Figure 6:**
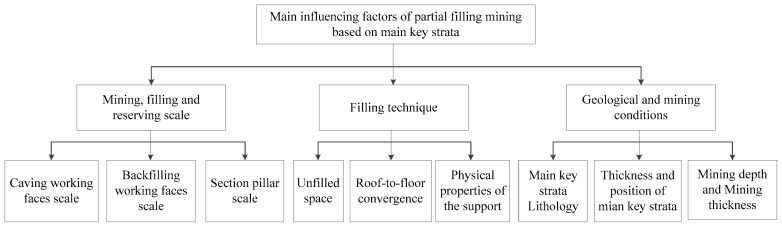
Surface subsidence caused by partial filling mining based on the main key stratum structure and the influencing factors of energy polling distribution control.

Coal mining-induced strata movement is a complicated problem that combines time and space. It takes place under the influence of multiple factors. It is determined by geological mining conditions (e.g., overlying rock lithology, mining depth, and mining thickness), mining mode, and mining space together. The strata movement and energy polling distribution control methods during local filling–caving multi-faces coordinated deep mining based on the main key stratum not only make full use of the control effect of the key stratum and advantages of partial filling mining but are also restricted by corresponding influencing factors. Based on the comprehensive analysis, the major factors could be divided into three types: (1) mining scale, filling scale, and section pillar scale; (2) filling technology; and (3) geological mining conditions (Figure 6).

(1) Mining scale, filling scale, and section pillar scale

The mining scale, filling scale, and section pillar scale had direct relations with coal yield, stability of the composite filling body, and overlying strata movement. They were major factors that influenced strata movement and energy polling distribution control.

(2) Filling technology

Filling technology factors include filling–roof space, roof-to-floor convergence, mechanical properties of the filling body and mechanical properties of the composite filling body. The filling–roof space refers to the spaces between the filling body and the roof, which was generally between 0 and 650 mm. The roof-to-floor convergence refers to the phenomenon when the roof and floor move toward the goaf due to the delayed filling after coal seam mining. It is generally within 100~400 mm. This might be relatively large for deep mining. The mechanical properties of the filling body and the composite filling body have a direct influence on the strata movement and energy polling distribution. Instead of lifting the gangues from the shaft, the proposed control methods of strata movement and energy polling distribution aim to digest gangues in the shaft while accomplishing the control effect. Hence, the proposed filling body in this study primarily referred to the gangues, whereas the composite supporting structure refers to the composite structure composed of gangues and section pillars. The gangue block size and cracking–swelling performances can significantly influence the mechanical properties of the gangues and the composite supporting structure.

(3) Geological mining conditions

The proposed geological mining conditions in this study include lithology, thickness, and height of the coal seam main key stratum, mining depth, and mining thickness. All of these factors have significant influences on strata movement and the energy polling distribution control methods of partial filling mining based on the main key stratum.

Based on the above analysis, influencing factors, such as geological mining conditions, could not be manually changed. Many filling technology factors could influence strata movement and energy polling distribution. However, they could influence the effective subsidence space of the roof. The influences of filling technology on surface subsidence control could be simulated by changing the filling ratio.

Hence, the influence of filling technology and mining–filling pillar scales on the control effects of the proposed method was investigated through a case study based on the Yingpanhao Coal Mine. The details (Table 6) are introduced as follows:

Moreover, the physical and mechanical parameters of the gangue filling zone determined with reference to previous studies [38] and model parameters (Table 7) were verified by the equivalence mining height principle [39].

## 4. Control Effects over Strata Movement and Energy Polling Distribution during Local Filling–Caving Multi-Faces Coordinated Deep Mining

### 4.1. Influences of Filling Ratio on Control Effects of Surface Subsidence and Energy Polling Distribution

In the paper, the FLAC3D 5.0 numerical simulation software was applied. The constitutive model of this numerical model (Table 8) was the Mohr–Coulomb model. The mechanical parameters of the strata in the model were determined from the mechanics experiment of the rock mass in the laboratory. The bottom boundary of the model was determined as u = v = w = 0 (u was the displacement in the x direction, v was the displacement in the y direction, and w was the displacement in the z direction). The top was the free boundary, and the left and right boundaries were fixed in the horizontal displacement.

To check the reliability of the model, we mined two working faces based on the actual situation of the Yingpanhao Mine. The working face was 300 m in width and 2000 m in length, and the two working faces were separated by the 300-m-wide coal pillar. Based on the measurement results of the surface, the subsidence of Point C52 was 326 mm, and the subsidence of the corresponding position in the numerical simulation was 350 mm. As one working face of the Yingpanhao Mine was 1800 m in length and the other was 1900 m in length, both of them were shorter than that of the working face in the numerical simulation. Thus, the simulation result was faintly larger than the actual subsidence value. The simulation results were basically consistent with the actual situation, and the established model was reliable.

**Figure 7:**
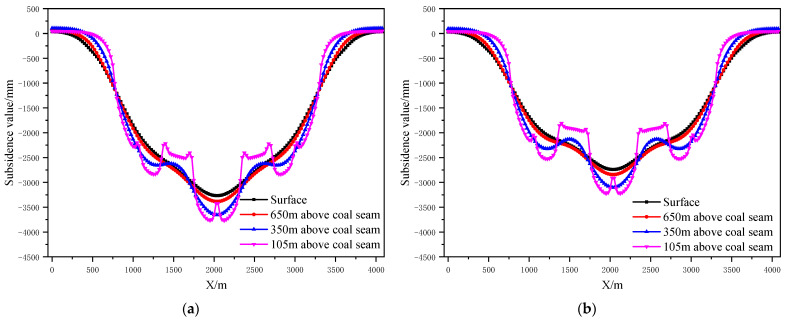

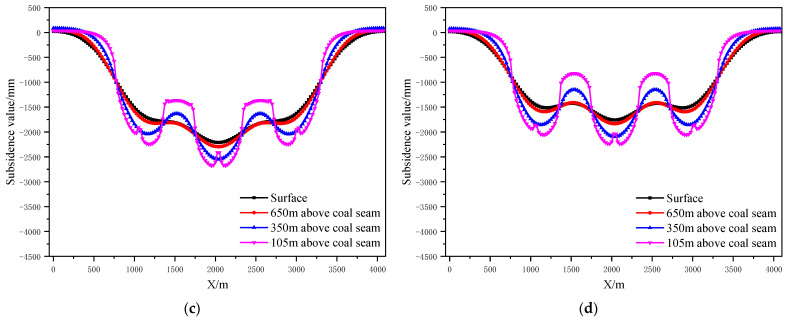
Strata and surface subsidence laws of partial filling mining based on the main key stratum under different filling ratios; (**a**) filling ratio = 60%. (**b**) Filling ratio = 70%. (**c**) Filling ratio = 80%. (**d**) Filling ratio = 90%.

**Figure 8:**
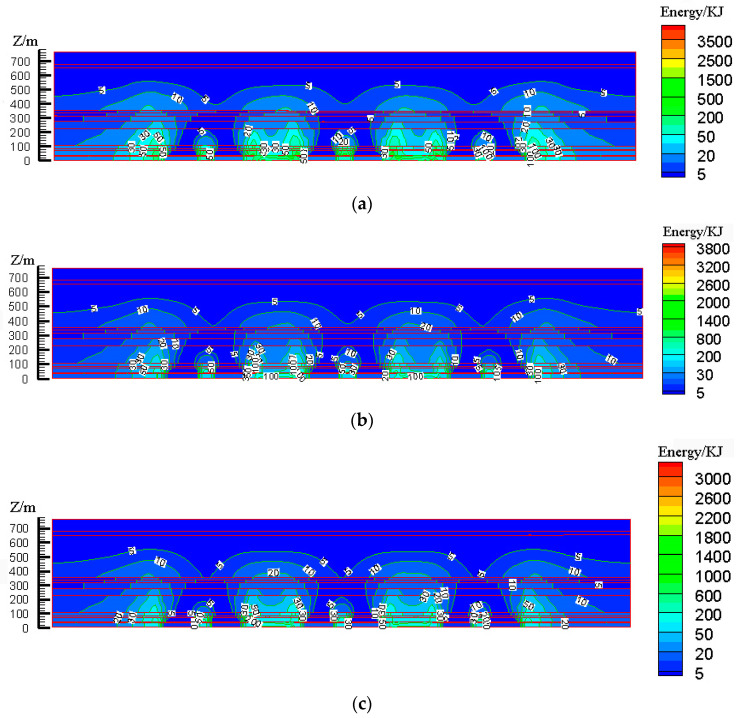

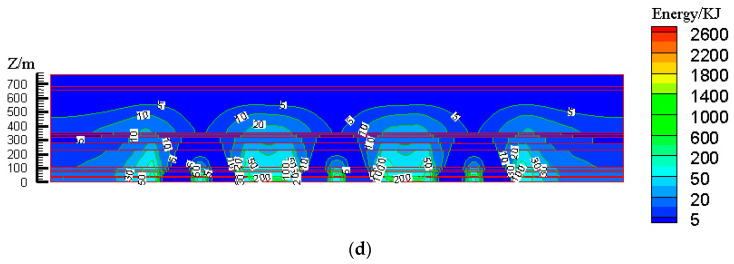
Effects of filling ratio on energy polling distribution characteristics of partial filling mining based on the main key stratum; (**a**) energy polling distribution characteristics of partial filling mining based on the main key stratum (filling ratio = 60%). (**b**) Energy polling distribution characteristics of partial filling mining based on the main key stratum (filling ratio = 70%). (**c**) Energy polling distribution characteristics of partial filling mining based on the main key stratum (filling ratio = 80%). (**d**) Energy polling distribution characteristics of partial filling mining based on the main key stratum (filling ratio = 90%).

According to the design scheme in Table 6, the FLAC3D numerical simulation software was applied. The corresponding strata and surface subsidence data, as well as the energy polling distribution values, were extracted. The corresponding subsidence curves and energy distribution diagrams were plotted (Figure 7 and Figure 8).

**Figure 9:**
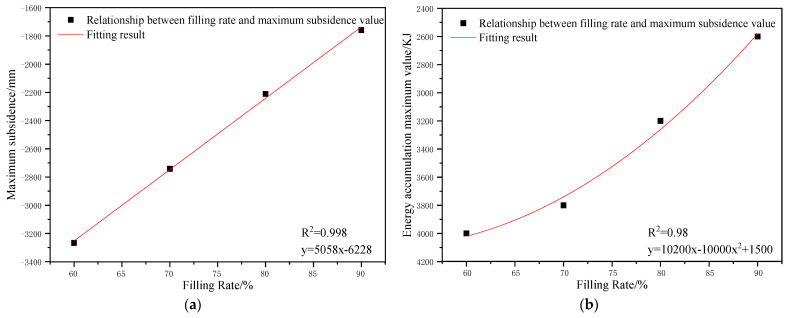
Influence of filling ratio on the control effect of partial filling mining based on the main key stratum; (**a**) relationship between filling ratio and maximum surface subsidence. (**b**) Relationship between filling ratio and energy accumulation maximum value.

To determine the influences of the filling ratio on the control effect of surface subsidence and energy polling distribution of partial filling mining based on the main key stratum, a statistical analysis of the maximum surface subsidence and maximum energy polling distribution values under different filling ratios was conducted. The corresponding relation curves were plotted. The results are shown in Table 9 and Figure 9.

Figure 9a indicated that the maximum surface subsidence decreased gradually with the increase in the filling ratio. A linear correlation between the maximum surface subsidence and the filling ratio was present with a linear correlation coefficient of R^2^ = 0.998. As shown in Figure 9b, the maximum energy polling distribution decreased gradually with the increase in the filling ratio. A parabolic correlation was present between the maximum energy polling distribution and filling ratio, with a correlation coefficient of R^2^ = 0.98.

As presented in Table 9, the filling ratio increased from 60% to 90%. The maximum surface subsidence decreased by 1509 mm, and the maximum energy polling distribution decreased by 1400 KJ, which indicated the significant influence of the filling ratio on surface subsidence and energy polling distribution. This could be attributed to the decline in the effective subsidence space of overlying strata with the increase in the filling ratio of partial filling mining. It was identical to the reduction in mining height in the equivalent mining height principle, which resulted in small variations of surface subsidence and energy polling distribution.

### 4.2. Influence of Mining-Filling-Pillar Scale on Control Effects of Surface Subsidence and Energy Polling Distribution

(1) Width of filling face

**Figure 10:**
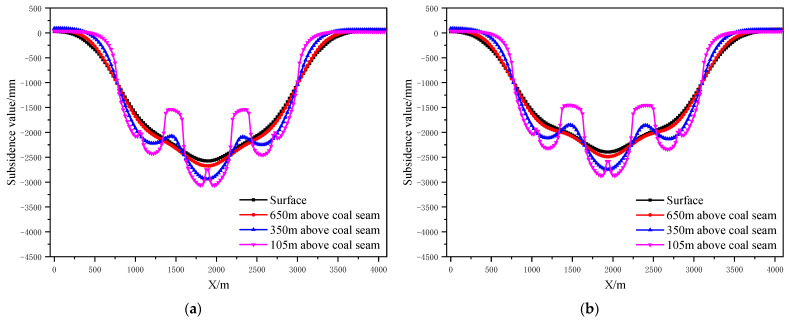

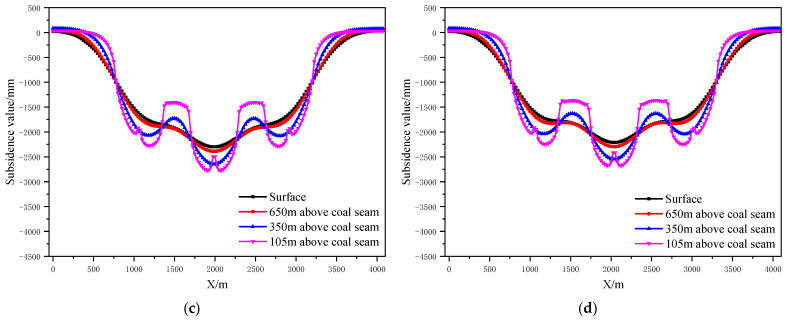
Strata and surface subsidence rule of partial filling mining based on the main key stratum under different filling face scales; (**a**) width of filling face =150 m. (**b**) Width of filling face =200 m. (**c**) Width of filling face =250 m. (**d**) Width of filling face =300 m.

**Figure 11:**
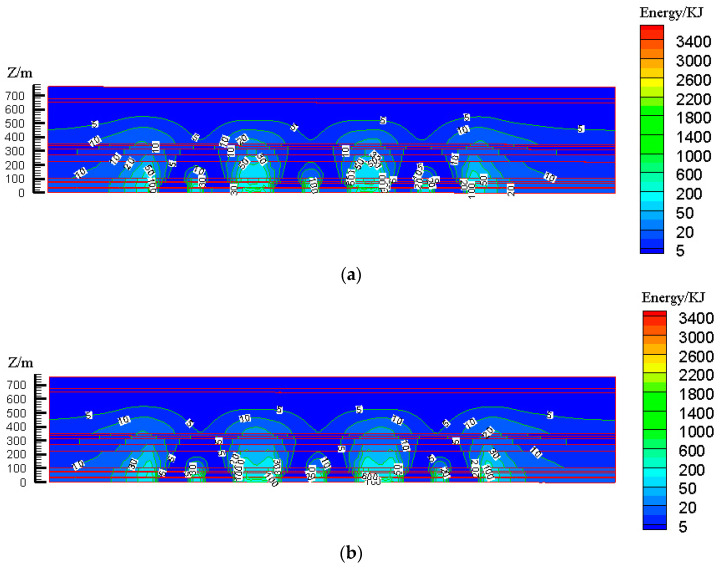

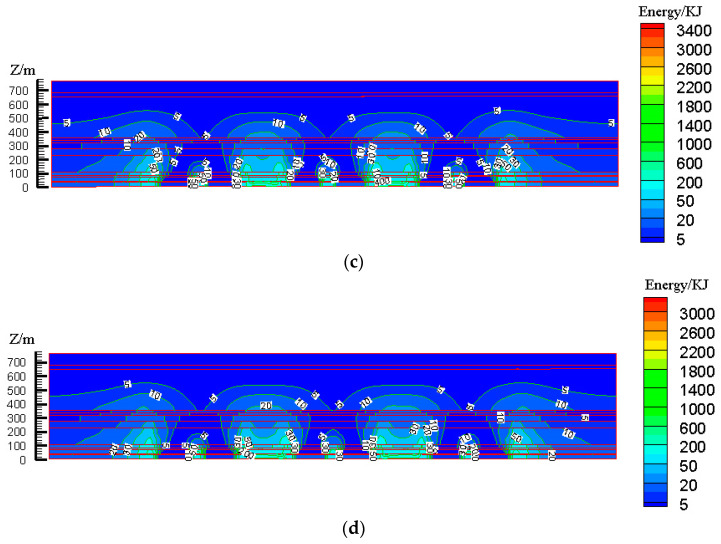
Influence of filling face scale on energy polling distribution characteristics of partial filling mining based on the main key stratum; (**a**) energy polling distribution characteristics of partial filling mining based on the main key stratum (width of filling face = 150 m). (**b**) Energy polling distribution characteristics of partial filling mining based on the main key stratum (width of filling face = 200 m). (**c**) Energy polling distribution characteristics of partial filling mining based on the main key stratum (width of filling face = 250 m). (**d**) Energy polling distribution characteristics of partial filling mining based on the main key stratum (width of filling face = 300 m).

According to the design scheme in Table 6, the FLAC3D numerical simulation software was applied. The corresponding strata, surface subsidence data, and energy polling distribution values at different buried depths were extracted. The corresponding subsidence curves and energy distribution diagrams were plotted (Figure 10 and Figure 11).

**Figure 12:**
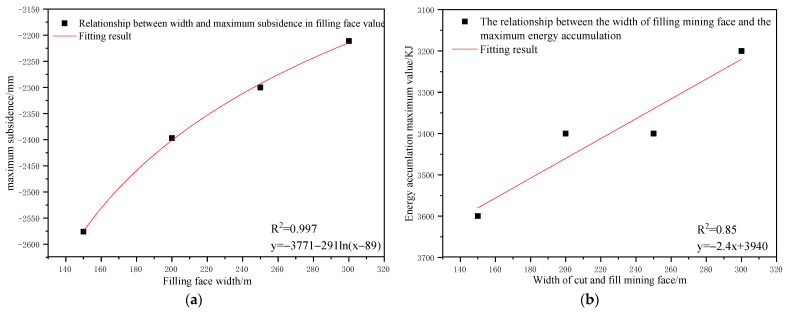
Influence of filling face scale on the control effect of partial filling mining based on the main key stratum; (**a**) relationship between filling face scale and maximum surface subsidence. (**b**) Relationship between filling face scale and energy accumulation maximum value.

To determine the influence of the filling face scale on the control effect of the surface subsidence and energy polling distribution of partial filling mining based on the main key layer, a statistical analysis of the maximum surface subsidence and maximum energy polling distribution values under different filling face scales was conducted. The corresponding relation curves were plotted. The results are shown in Table 10 and Figure 12.

As shown in Figure 12a, the maximum surface subsidence decreased gradually with the increase in the filling face scale. A logarithmic correlation between the maximum surface subsidence and filling face scale was present with a correlation coefficient of R^2^ = 0.997. As shown in Figure 12b, the maximum energy polling distribution presented a reduction trend with the increase in the filling face scale. A linear correlation between the maximum energy polling distribution and filling face scale was present with a correlation coefficient of R^2^ = 0.85.

As shown in Table 10, the width of the filling face increased from 150 m to 300 m, the maximum surface subsidence decreased by 365 mm and the maximum energy polling distribution decreased by 400 KJ. With an increase in the filling face scale, the mutual response degree of goaves at two sides of the face decreases gradually. Moreover, the mutually independent incomplete mining spaces were formed, accompanied by a gradual reduction in the surface mining and the overlying strata loads on the section pillar. Compared to other factors, the width of the filling face had minimal impact on the surface subsidence and energy polling distribution. Therefore, it was not a major influencing factor.

(2) Width of caving face

**Figure 13:**
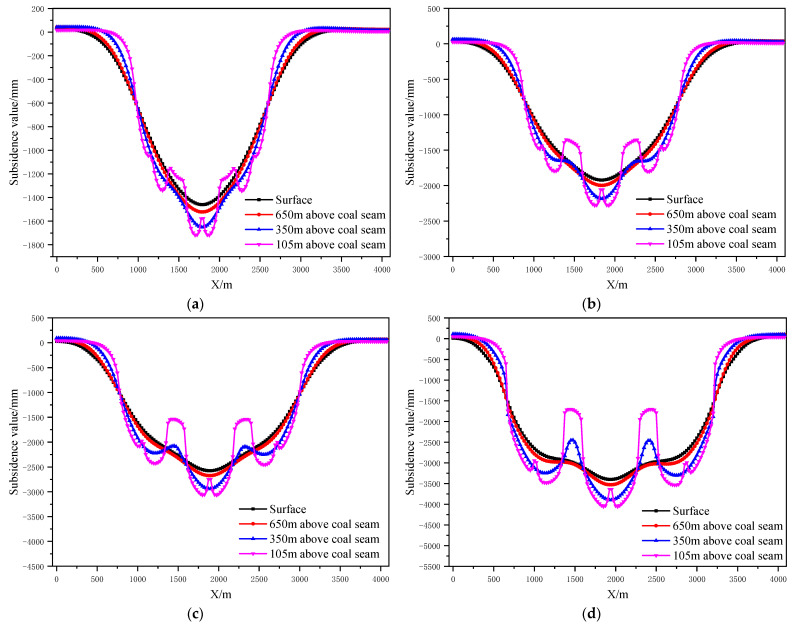
Strata and surface subsidence rule of partial filling mining based on the main key stratum under different caving face scales; (**a**) width of caving face = 200 m. (**b**) Width of caving face = 250 m. (**c**) Width of caving face = 300 m. (**d**) Width of caving face = 350 m.

**Figure 14:**
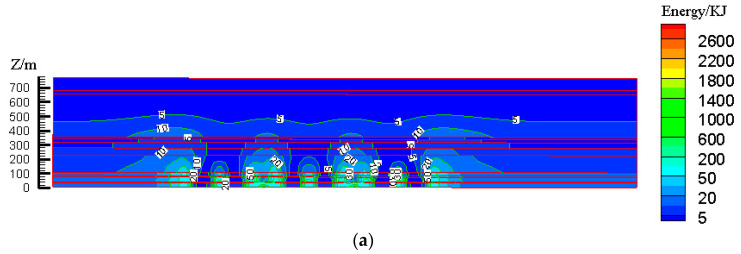

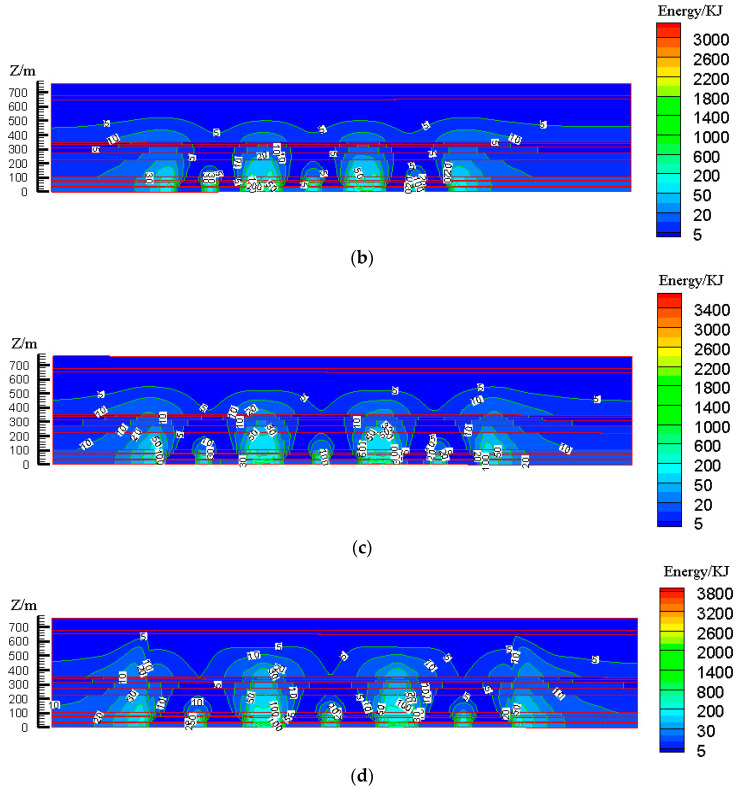
Influence of caving face scale on energy polling distribution characteristics of partial filling mining based on the main key stratum; (**a**) width of caving face = 150 m, (**b**) width of caving face = 200 m, (**c**) width of caving face = 250 m, and (**d**) width of caving face = 300 m.

According to the design scheme in Table 6, the FLAC3D numerical simulation software was applied. The corresponding strata, surface subsidence data and energy polling distribution values at different buried depths were extracted. The corresponding subsidence curves and energy distribution diagrams were plotted (Figure 13 and Figure 14).

**Figure 15:**
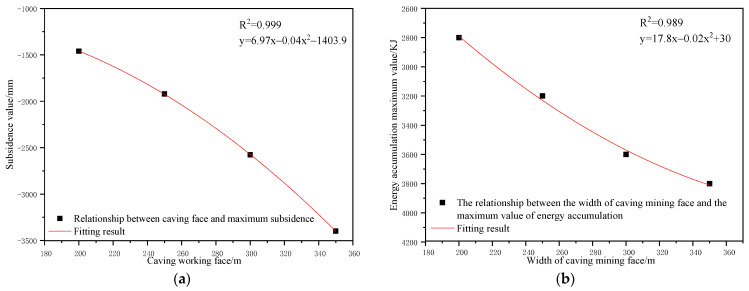
Influence of caving face scale on control effect of partial filling mining based on the main key stratum; (**a**) relationship between caving face scale and maximum surface subsidence. (**b**) Relationship between caving face scale and maximum energy polling distribution value.

To determine the influences of the caving face scale on the control effect of the surface subsidence and energy polling distribution of partial filling mining based on the main key stratum, a statistical analysis of the maximum surface subsidence and maximum energy polling distribution values under different caving face scales was conducted. The corresponding relation curves were plotted. The results are shown in Table 11 and Figure 15.

As shown in Figure 15a, the maximum surface subsidence increased gradually with increasing caving face scale. A parabolic correlation was present between the maximum surface subsidence and the caving face scale according to Origin fitting results (R^2^ = 0.999). As shown in Figure 15b, the maximum energy polling distribution presented an increasing trend with the increase in caving face scale. A parabolic correlation between the maximum energy polling distribution and the caving face scale was present (R^2^ = 0.989).

As presented in Table 11, the width of the caving face increased from 200 m to 350 m, the maximum surface subsidence increased by 1939 mm and the maximum energy polling distribution increased by 1000 KJ, which indicated a significant change. With the increase in the caving face scale, the mining degree of the goaf increased significantly. Moreover, the failure height of overlying strata rapidly increased, accompanied by fast expansion of the failure range. In addition, the mutual influencing degrees of adjacent goaves increased and the surface mining degree significantly increased. The overlying strata loads on the section pillar intensified dramatically. Compared to other factors, the width of the caving face significantly influenced surface subsidence and energy polling distribution. Therefore, it was considered a major influencing factor.

(3) Section pillar width

**Figure 16:**
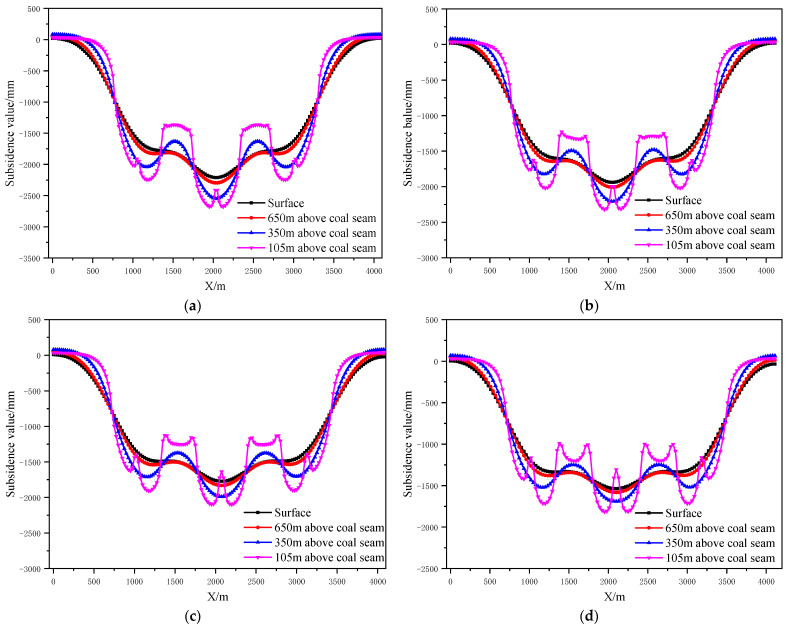
Strata and surface subsidence rule of partial filling mining based on main key stratum in different section pillar scale; (**a**) With of section pillar = 25 m. (**b**) With of section pillar = 30 m. (**c**) With of section pillar = 50 m. (**d**) With of section pillar = 60 m.

**Figure 17:**
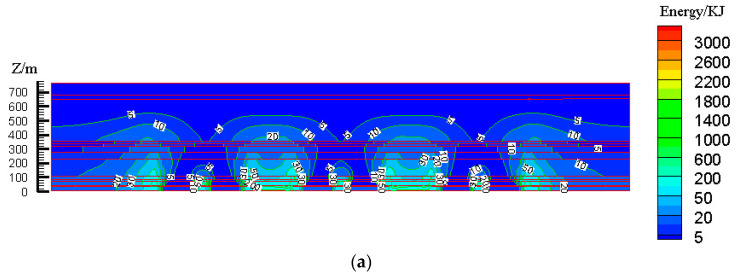

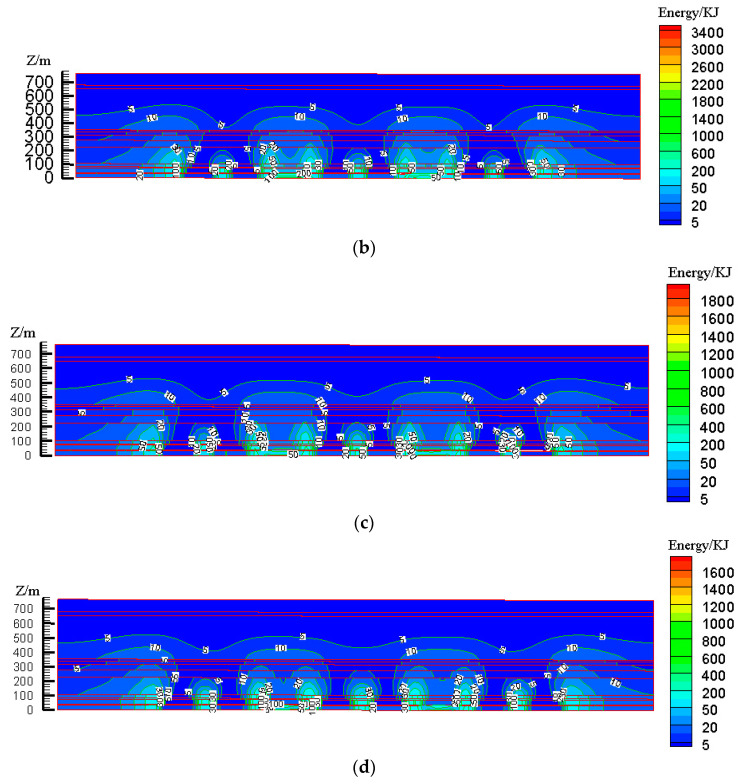
Influence of section pillar scale on energy polling distribution characteristics of partial filling mining based on the main key stratum; (**a**) Width of section pillar = 25 m, (**b**) Width of section pillar = 30 m, (**c**) Width of section pillar = 50 m and (**d**) Width of section pillar = 60 m.

According to the design scheme in Table 6, the FLAC3D numerical simulation software was applied. The corresponding strata and surface subsidence data and energy polling distribution values at different buried depths were extracted. The corresponding subsidence curves and energy distribution diagrams were plotted (Figure 16 and Figure 17).

**Figure 18:**
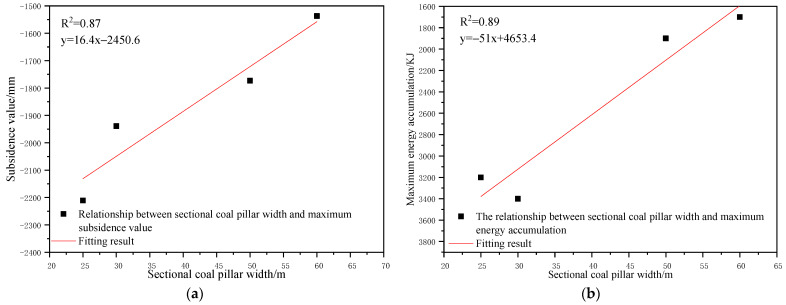
Influence of sectional coal pillar width on control effect of partial filling mining based on main key stratum; (**a**) Relationship between sectional coal pillar width and maximum subsidence value. (**b**) Relationship between sectional coal pillar width and maximum energy accumulation.

To determine the influence of the section pillar scale on the control effect of surface subsidence and energy polling distribution of partial filling mining based on the main key stratum, a statistical analysis of the maximum surface subsidence and maximum energy polling distribution values under different section pillar scales was conducted. The results are shown in Table 12 and Figure 18.

As shown in Figure 18a, the maximum surface subsidence decreased gradually with the increase in the section pillar scale. A linear reduction correlation between the maximum surface subsidence and section pillar scale was present according to Origin fitting results (R^2^ = 0.87). As shown in Figure 18b, that the maximum energy polling distribution decreased gradually with the increase in the section pillar scale. According to the Origin fitting results, a linear reduction between the maximum energy polling distribution and section pillar scale was present with a correlation coefficient of R^2^ = 0.89.

As present in Table 12, the section pillar width increased from 25 m to 60 m, the maximum surface subsidence decreased by 674 mm, and the maximum energy polling distribution increased by 1500 KJ. The latter changed dramatically, whilst the former underwent just minor change. With the increase in the section pillar scale, the section pillar further restricted the upward development of overlying strata failure. As a result, the failure range decreased gradually. Moreover, the mutual influencing degrees of adjacent goaves decreased to some extent and the surface mining degree decreased slightly. Compared to other factors, section pillar width had a minor impact on the surface subsidence, and hence, was considered a secondary influencing factor. However, it significantly influenced the energy polling distribution. Therefore, it was considered a major influencing factor.

### 4.3. Comprehensive Analysis of Strata Movement and Energy Polling Distribution Control Factors of Partial Filling Mining Based on the Main Key Stratum

Based on the above analysis, the strata movement and energy polling distribution were influenced by the filling ratio, caving face width, filling face width, and section pillar width. A comprehensive analysis was required to determine the order of these influencing factors. The range standardization method was used to execute dimensionless treatment of factors in order to eliminate differences in units among the different influencing factors. The parameters were then transformed into numerical values within 0~1. The range standardization formula was as follows:

(1) $$x_{j}^{\prime }=\frac{x_{i}-min_{x_{i}}}{max_{x_{i}}-min_{x_{i}}}.$$

**Figure 19:**
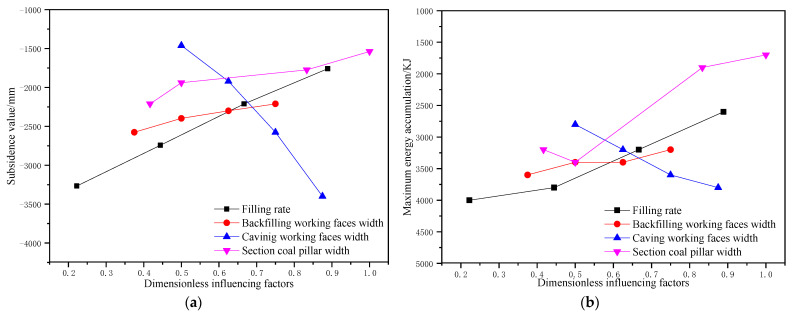
Influence of dimensionless factors on strata movement and energy polling distribution; (**a**) surface subsidence. (**b**) Energy polling distribution.

Before the dimensionless treatment of factors, it was necessary to determine the ranges of the factors. With reference to the relevant cases of deep long-walled caving mining, the width of the working face could reach 400 m at most. Therefore, the range of the caving face width, the filling face width, and the section pillar scale were 0~400 m, 0~400 m, and 0~60 m, respectively. Considering the limitation of the current filling mining technology, the filling ratio of the working face ranged between 50% and 95%. After dimensionless treatment of the influencing factors, the corresponding control effect relation curves were plotted (Figure 19).

As shown in Figure 19, premising of no serious surface failure and strata pressure behaviors, the order of the influencing factors on the surface subsidence of partial filling mining based on the main key stratum was as follows: width of caving face > filling ratio > section pillar width > width of filling face. The order of the influencing factors on the energy polling distribution of partial filling mining based on the main key stratum was as follows: section pillar width > filling ratio > caving face > width of filling face.

## 5. Superiority Analysis of Local Filling–Caving Multi-Face Coordinated Deep Mining

To verify the superiority of partial filling mining based on the main key stratum, the degree of surface subsidence and overlying strata failure was simulated during full caving mining, complete filling mining, wide strip mining, mixed filling mining, large mining (small reserved width), and partial filling mining. The corresponding mining schemes are listed in Table 13.

**Figure 20:**
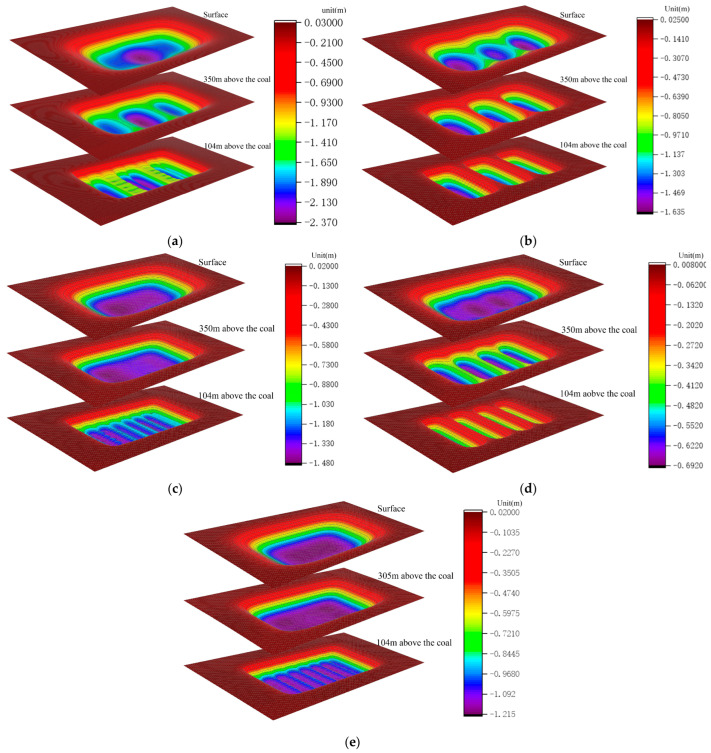
Temporal and spatial evolutionary laws of subsidence spaces; (**a**) partial filling mining. (**b**) Large mining width, small reserved width. (**c**) Mixed filling mining. (**d**) Wide stripe mining. (**e**) Full filling mining. Note that the calculated data were extracted from FLAC3D 5.0 and imported to Origin2018 to draw the subsidence diagrams of rock strata with different buried depths, respectively. Then, the common legend was obtained by the same method. Final, the above subsidence diagrams and legend were imported to Photoshop 2018, and one of the pictures in Figure 20 can be obtained after the typesetting.

The corresponding three-dimensional numerical models were constructed according to the mining schemes in Table 13. The strata subsidence values at different buried depths were extracted and used to plot the temporal, as well as the spatial, evolutionary laws of the corresponding subsidence space (Figure 20).

**Figure 21:**
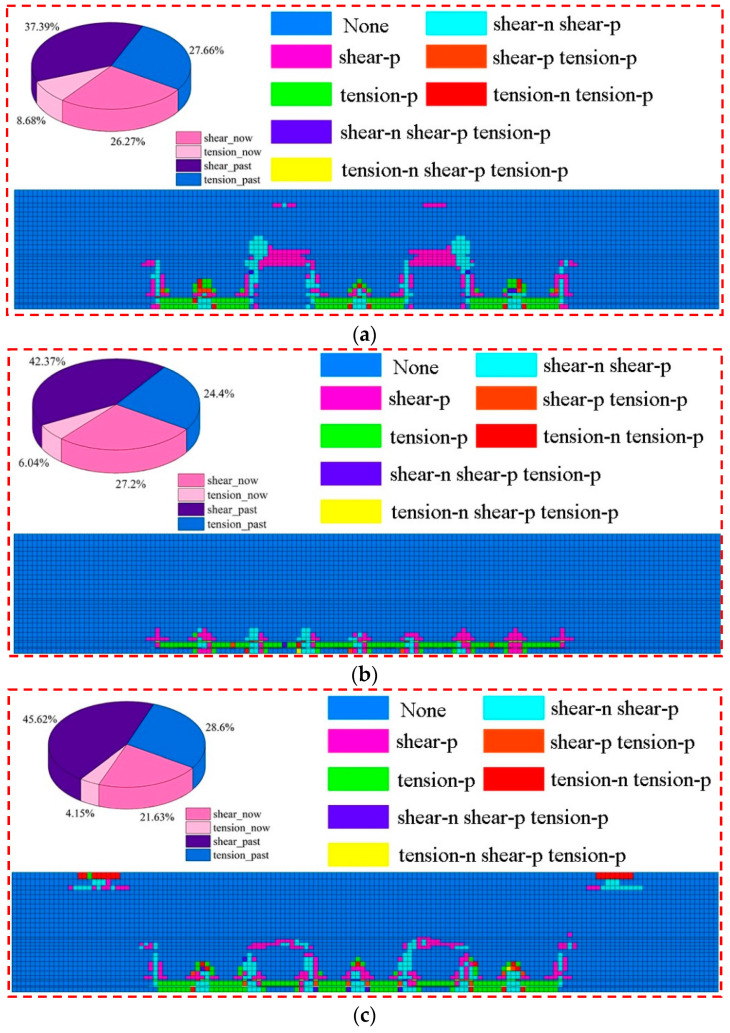

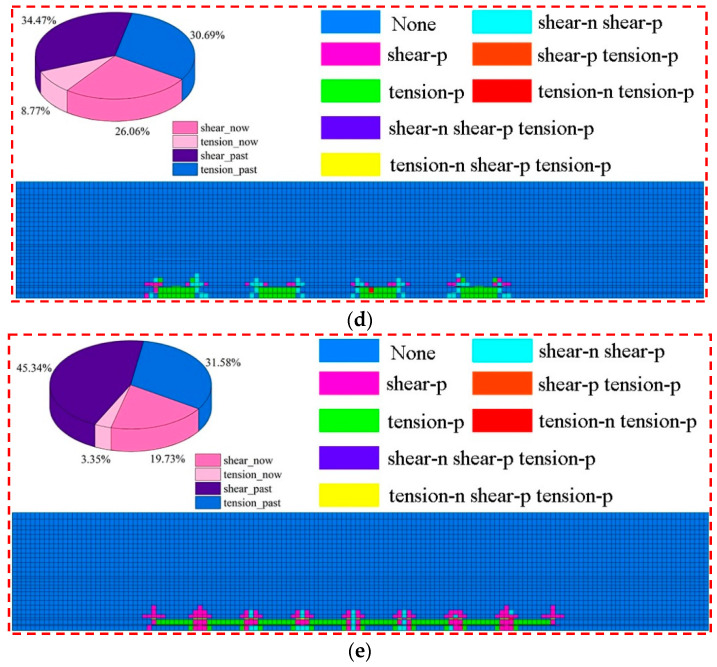
Overlying strata failure degrees of different mining modes; (**a**) large mining width small reserved width. (**b**) Mixed filling mining. (**c**) Local filling–caving multi-face coordinated mining. (**d**) Wide stripe. (**e**) Full filling mining.

The program for extracting overlying strata failure volume was developed based on the Fish language, which was used to extract overlying strata failure volume and energy polling distribution values in the corresponding numerical model. The overlying strata failure diagram is shown in Figure 21. As shown in Figure 21a, tensile failure accounts for 36.34% of the overburden failure volume, while shear failure accounts for 63.66%. A large area of shear failure was present above the coal pillar working face. Failure height did not develop up to the water barrier. As shown in Figure 21c, tensile failure accounts for 32.75% of the overburden failure volume, while shear failure accounts for 67.25%. A large area of shear failure was present above the backfilling working face. Failure height did not develop up to the water barrier. On both sides of the goaf, tensile failure development was observed near the surface.

In Figure 21, the failure pattern of the overlying strata is saddle-shaped in mixed filling mining, wide stripe and full filling mining. The stress concentrated near the coal pillar and the damage area is also near the coal pillar. In large mining width small reserved width, the failure area mainly occurs at the coal wall of the goaf, and shear failure occurs in tens of meters above wide coal pillar. The overall failure pattern shows a regular broken line. In addition, due to the influence of rock dislocation, shear failure also occurs in some areas on the surface of Zhidan Group sandstone. In local filling–caving multi-face coordinated mining, the failure area mainly occurs at the coal wall of the goaf. Shear failure also occurred in some strata above the filling working face, and the failure form was arching. In addition, tensile failure occurred near the surface on both sides of the mining area. This is because the phenomenon of tensile stress concentration will occur at this location when the mining area is large and the rock strata move violently to the goaf.

For the intuitive analysis of the surface subsidence and strata failure under different mining modes, the corresponding filling ratio, yield rate, coal pillar ratio, maximum surface subsidence volume, maximum energy polling distribution, and overlying strata failure volume were extracted and calculated (Table 14).

As present in Table 14, the surface subsidence reduction effect followed the trend as wide strip mining > full filling mining > large mining width small reserved width > mixed filling mining > partial filling mining based on the main key stratum > full caving mining. Considering the energy polling distribution control effect, the trend was as follows: wide strip mining > large mining width small reserved width > full filling mining = mixed filling mining > partial filling mining based on the main key stratum > full caving mining. If the yield rate and coal pillar ratio reflect coal utilization, the area filling ratio reflects the coal mining cost indirectly and the strata failure rate reflects the degree of ecological environmental damages to some extent. Considering the groundwater protection effect, the trend was follows: wide strip mining > full filling mining > mixed filling mining > large mining width small reserved width > partial filling mining based on the main key stratum > full caving mining. Comprehensive analysis revealed that mixed filling mining and partial filling mining based on the main key layer performed best in terms of cost. The simultaneous mining and filling on the same working face was made possible by mixed-filling mining even if it had significant technological challenges. Therefore, partial filling mining based on the main key stratum was the preferred mining mode while having the highest cost-effectiveness in surface subsidence control.

**Figure 22:**
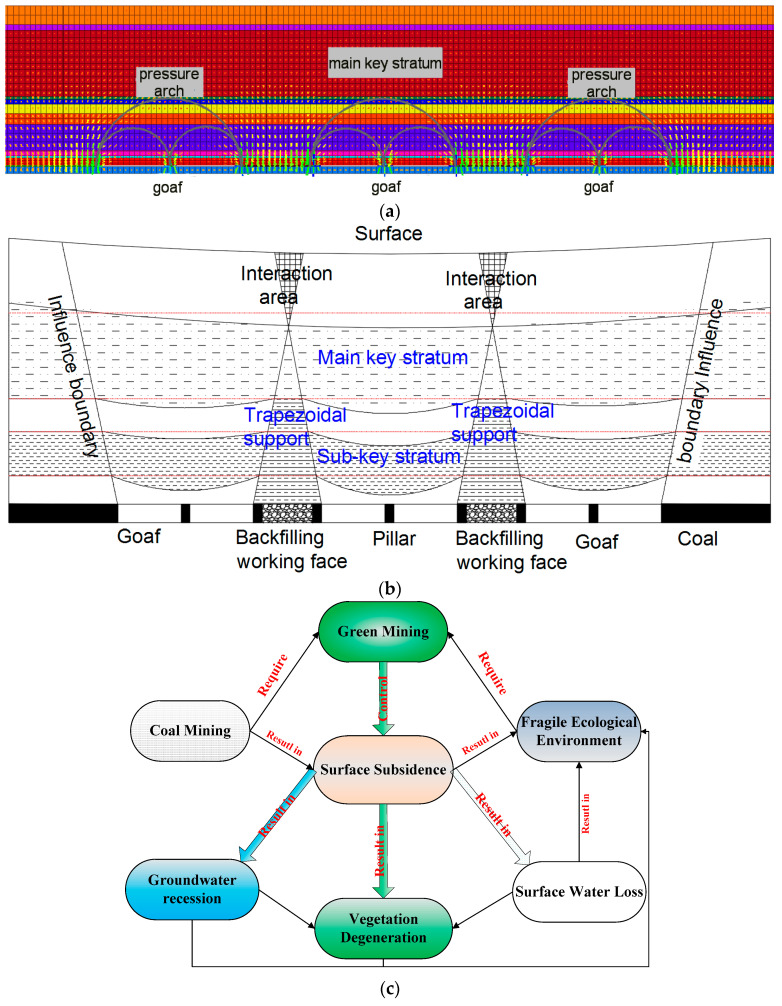
Control mechanism of the local filling–caving multi-faces coordinated mining; (**a**) distribution diagram of pressure arch. (**b**) Diagram of subsidence space transfer [50]. (**c**) Logic diagram of research content and geological environment.

In order to further explain the superiority of this control method, the control mechanism of this method is also described briefly (Figure 22). The composite filling structure formed by the filling working face and the section coal pillar divides the entire mining area into three independent insufficient mining spaces, and acts as a wide isolated coal pillar to support the overburden load. A caving fractured zone was forming after a single working face mined, and the upper rock mass breaks and collapses. The sub-critical stratum restricts the caving fractured zone from continuing to develop upward. A pressure arch in the middle-low part of the sub-key stratum was formed when the load of the sub-key stratum and its overlying strata was transferred to both sides and concentrated. A large stress arch under the main key stratum is formed when two consecutive working faces are mined, and the load of the main key stratum and its overlying strata is transferred to both sides and concentrated on the coal walls of both sides of the goaf.

In conclusion, the composite support and main key strata (main key strata and sub-key strata) forms a dual control system of coordinated deformation, which step by step realizes the control of the movement of the overlying strata: the sub-key strata is close to the coal seam, which directly limits the damage height of the overlying strata and reduces the effective sinking space for upward transmission. The chief key stratum limits the upward transmission of the wave-shaped sinking basin, causing the overlying stratum to be a single gently sinking basin.

Through studying the strata movement control method in this paper, we can reduce the damage of coal mining to geological environment and realize green mining on the premise of ensuring efficient and safe coal mining (Figure 22c).

## 6. Conclusions

A local filling–caving multi-faces coordinated mining approach based on the main key stratum was proposed as a strata movement control method after the investigation of subsidence and overlying strata structural characteristics in the Yingpanhao Coal Mine. Relevant influencing factors and influencing laws were explored. Moreover, the superiority of this method was investigated from numerous perspectives, such as overlying strata failure and surface damage degree. Some major conclusions could be drawn as follows:

(1) The maximum subsidence velocity at monitoring points during the single working face of the Yingpanhao Coal Mine was 1.24 mm/d, which was lower than the limit (1.67 mm/d) in the active phase determined in the Guideline for Buildings, Water Body, Railway and Major Roadway Coal Pillar Setting and Coal Mining. Additionally, prediction parameters, which mainly influence angular tangent and offset of deflection points, were within the reference ranges. However, the subsidence factor, which was a key prediction parameter, was only 47.5% of the lower limit of the reference range.

(2) Although sandstones in the Zhidan Group were relatively soft and there was rich porosity, most bonding materials had calcite cementation and a few clay minerals. Cement composition was almost consistent with detritus, which did not disintegrate in water. The strata were found to be relatively thick, while hardly developed fractures and vertical joints. There were almost no faults or folds. The strata generally had great rigidity and presented very strong control effects. They made up the key stratums in the overlying rock structure.

(3) The influences of the filling ratio, caving face scale, filling face scale, and section pillar scale on the control effects of strata movement and energy polling distribution were analyzed. The results show that with the increase in filling ratio, surface subsidence presented a linear functional reduction trend and energy polling distribution presented a parabolic functional reduction trend. With the increase in the filling face width, surface subsidence presented a logarithmic functional reduction and energy polling distribution presented a linear functional reduction. With the increase in caving face width, surface subsidence and energy polling distribution both presented parabolic functional growths. With the increase in section pillar scale, the surface subsidence and energy polling distribution presented a linear functional reduction trend.

(4) Relative influencing degrees of factors on the control effects of strata movement and energy polling distribution during partial filling mining based on the main key stratum were analyzed by the range standardization method. The following criteria were ranked from high to low in terms of their ability to restrict strata movement while the main key stratum could still bear overlying strata loads: caving face width > filling ratio > section pillar width > filling face width. The influencing factors of the energy polling distribution were also list in order from high to low degrees as follows: section pillar width > filling ratio > caving face > filling face width.

(5) Control effects of strata movement and energy polling distribution in full caving mining, full filling mining, wide strip mining, and mixed filling mining were investigated by numerical simulation. Considering the surface subsidence reduction effect, the factors were ranked as wide strip mining > full filling mining > large mining width small reserved width > mixed filling mining > partial filling mining based on the main key stratum > full caving mining. Considering the groundwater protection effect, the factors were ranked as wide strip mining > full filling mining > mixed filling mining > large mining width small reserved width > partial filling mining based on the main key stratum > full caving mining. With comprehensive considerations, partial filling mining based on the main key stratum demonstrated the highest cost-effectiveness.

## Figures and Tables

**Table 1:** Inversion results of strike line subsidence data when working face advances to 1634 m.

	Subsidence Coefficient	Tangent of Main Influence Angle	Propagation Angle of Mining Impact/°	Inflection Point Offset Coefficient	Mean Square Error/mm
2201 propulsion 1634 m	0.171	1.13	89.5	0.08	9.6
Reference value range	0.36~0.55	0.96~1.20	—	0.04~0.15	—

**Table 2:** Surface subsidence factors in the east and west mining wells.

Working Face No.	Working Face Width/m	Length of Working Face/m	Mining Thickness/m	Burial Depth/m	Topsoil Layer/m	Bedrock Thickness/m	Subsidence Coefficient q
Daliuta live well 12,205	230	2251	3.50	110	22	88	0.73
Liuta Mine 12,106	246.8	633	6.90	151	30	120.6	0.77
Fenjiata 1201	250	1850	3.30	147	10	137	0.75
Cuncaota 22,111	224	2085	2.80	249	8	240.9	0.68
Buertai Mine 22,103-1	360	4250	2.90	292	19	273	0.64
Cuncaota No.2 Mine 22,111	300	3648	2.90	305	15	294	0.68
Xiaojihan Coal Mine 11,203	240	2245	2.67	350	25	315	0.60
Bayan Gaole Coal Mine 311,101~311,103	810	2600	5.30	650	118	532	0.44
Narin River 2 31,101	240	3030	5.50	650	78	572	0.16
Yingpanhao coal mine 2201	300	1800	6.00	725	45	680	0.08
Tangkou Coal Mine 1302	210	1560	3.64	960	212	748	0.53
Tangkou Coal Mine 1301	215	1320	3.38	1000	212	788	0.52
Tangkou Coal Mine 1307~1302	420	1440	7.02	980	212	768	0.65
Tangkou Coal Mine 1304	150	1457	4.97	910	212	698	0.44
Tangkou Coal Mine 1305	130	1540	4.97	920	212	708	0.32
Tangkou Coal Mine 2307~2308	420	1350	3.60	865	212	653	0.78
Tangkou Coal Mine 2307	210	1320	3.61	865	212	653	0.53
Tangkou Coal Mine 4305	120	1255	3.12	1040	212	828	0.45
Tangkou Coal Mine 4304~4305	240	1209	3.12	1060	212	848	0.50
Tangkou Coal Mine 2307~2310	825	1263	4.10	825	212	613	0.84
Tangkou Coal Mine 5301~5303	510	1541	4.80	965	212	753	0.70
Dongping Coal Mine 15,412	150	258	6.82	200	10	190	0.90
Xuchang Coal Mine 1315	163	1220	5.60	278	200	78	0.85
Daizhuang Coal Mine 1303	160	1300	2.90	400	245	155	0.63
Daizhuang Coal Mine 2301	150	650	2.90	440	245	195	0.71
Anju Coal Mine2302	100	770	2.50	895	227	668	0.04

**Table 3:** Strata distribution of deep mining area in the part of Dongsheng coalfield.

Stratum			Yingpanhao Coal Mine	Bayan Gaole Coal Mine	Nalinhe No.2 Well Coal Mine
System	Series	Formation	Thickness/m		
Quaternary	Holocene series	Alluvial proluvial sand	45.72–123.61 86.41	73.92–161.60 118.74	49.38–83.84 65.56
		Aeolian deposit			
	Upper Pleistocene	Malan Formation			
Cretaceous system	Zhidan Group		253.04–429.91 347.77	104.46–255.88 178.67	53.88–279.08 138.31
Jurassic (Main coal bearing strata)	Middle series	Diazepam group	50.9–134.80 94.66	35.90–157.29 96.60	67.53–154.53 103.15
		Zhiluo Formation	126.26–229.34 169.72	71.12.00–238.60 154.86	111.92–199.34 151.44
	Middle Lower Series	Yan’an Formation	307.80–393.14 355.07	208.67–312.28 260.47	334.32–365.30 349.81

**Table 4:** Strata distribution of deep mining area in the part of Central-Eastern China.

Stratum				Anju Coal Mine	Tangkou Coal Mine		Jining No.2 Well Coal Mine
System	Series	Formation		Thickness/m			
Quaternary				219.05–246.30 227.57	185.00–228.50 212.55		149.40–250.00 188.35
Jurassic	Upper series	Three sets		441.20–1113.65 690.50 56.90–186.70 124.05 (Magmatic rock)	391.163.60–125.20 70.26 (Magmatic rock)		285.170.00–154.79 96.96 (Magmatic rock)
Permian (Coal bearing strata)	Upper series	Upper Shihezi Formation		83.65–244.40 161.39	305.87		116.29
	Lower Series	Lower Shihezi Formation		37.50–57.6 48.59			15.06–90.00 55.17
		Shanxi Formation		61.90–102.49 75.29	87		59.97–118.10 93.68
Carboniferous (Coal bearing strata)	Upper series	Taiyuan Formation		36.01–46.53 40.22	157.35–188.37 168.00		145.35–196.50 170.35
	Middle series	Benxi Formation		—	4.00–34.42 17.22		43.00–78.99 66.37
Fault development	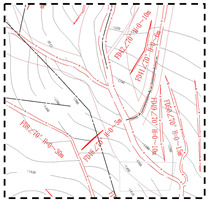 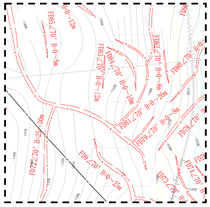		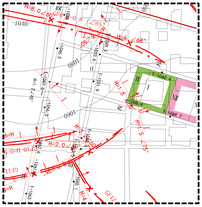 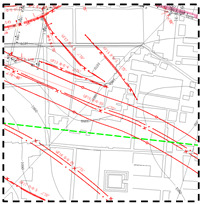			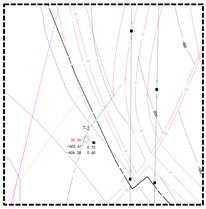 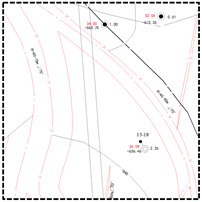	
	Anju Coal Mine		Tangkou Coal Mine			Jining No.2 Well Coal Mine	

**Table 5:** Quantitative analysis table of total rock X-ray diffraction.

Quartz/%	Potash Feldspar/%	Plagioclase/%	Calcite/%	Dolomite/%	Hematite/%	TCCM/%
54.1	11.5	18.4	5.1	2.4	0	8.5
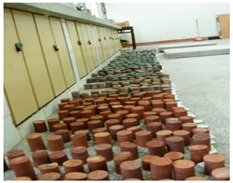		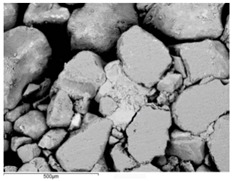		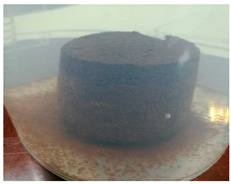		
Shear test		3D electron microscopy scanning experiments		total water immersion test		

**Table 6:** Study scheme of the effect of filling ratio on surface subsidence and energy polling.

Isolated Coal Pillar/m	Width of Caving Face/m	Width of Filling Working Face/m	Filling Rate/%	Length of Strike/m
25	300	300	90	2490
			80	
			70	
			60	
25	300	150	80	2490
		200		
		250		
		300		
25	200	150	80	2490
	250			
	300			
	350			
25	300	300	80	2490
30				
50				
60				

**Table 7:** Physical and mechanical parameters of the filling body.

Mechanical Parameters	Bulk Modulus/GPa	Shear Modulus/GPa	Friction Angle/°	Cohesion/MPa	Density /kg·m^−3^	Poisson’s Ratio
coal seam	1.35	0.587	6	8.89	1210	0.31
Gangue filling area	0.21	0.095	28	2	1500	0.3

**Table 8:** Three-dimensional numerical model [49].

Strata	Thickness/m	Diagram
Loess	86	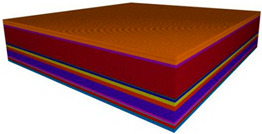 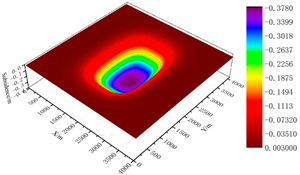
Sandy mudstone 5	27	
Zhidan group sandstone	300	
Coarse sandstone	14	
Sandy mudstone 4	22	
An’ding group sandstone	40	
An’ding-zhiluo group sandstone	50	
Zhiluo group sandstone	120	
Sandy mudstone 3	23	
Medium sandstone 1	10	
Sandy mudstone 2	33	
Coal	6	
Sandy mudstone 1	32	

**Table 9:** Statistics of maximum surface subsidence and maximum energy polling distribution under different filling ratios.

Fixed Parameters	Filling Rate/%	Maximum Subsidence Value/mm	Maximum Energy Accumulation /KJ
Width of caving mining face 300 m, width of filling mining face 300 m and Section coal pillar width 25 m	60	−3267	4000
	70	−2742	3800
	80	−2211	3200
	90	−1758	2600

**Table 10:** Statistical table of surface subsidence and energy polling extreme values in different filling face scale.

Fixed Parameters	Width of Filling Face/m	Maximum Subsidence Value/mm	Maximum Energy Accumulation/KJ
The width of caving mining face is 300 m, the filling rate is 80%, and the width of section coal pillar is 25 m	150	−2576	3600
	200	−2397	3400
	250	−2300	3400
	300	−2211	3200

**Table 11:** Statistics of maximum surface subsidence and maximum energy polling distribution under different caving face scales.

Fixed Parameters	Width of Caving Mining Face/m	Maximum Subsidence Value/mm	Maximum Energy Accumulation/KJ
The width of filling mining face is 150 m, the filling rate is 80%, and the width of section coal pillar is 25 m	200	−1460	2800
	250	−1921	3200
	300	−2576	3600
	350	−3399	3800

**Table 12:** Statistical table of surface subsidence and energy polling extreme values in different section pillar scale.

Fixed Parameters	Section Coal Pillar Width/m	Maximum Subsidence Value/mm	Maximum Energy Accumulation/KJ
Width of filling caving mining face: 300 m Filling rate 80%	25	2211	3200
	30	1939	3400
	50	1773	1900
	60	1537	1700

**Table 13:** Scheme designs of different mining modes.

Mining Method	Caving Working Face Width/m	Filling Working Face Width/m	Filling Rate/%	Advance Length of Strike/m	Section Coal Pillar/m
Full caving mining	300	0	0	2520	30
Full filling	0	300	80	2520	30
Wide strip mining	300	0	0	2520	30
Mixed filling mining	300	300	80	2520	30
Large mining width small reserved width	630	0	0	2520	30
Partial filling mining based on main key strata	630	300	80	2520	30

**Table 14:** Statistics on maximum surface subsidence and maximum energy polling distribution under different mining modes.

Mining Method	Maximum Subsidence/mm	Maximum Energy/KJ	Subsidence Coefficient	Filling Rate/%	Mining Out Rate/%	Coal Column Rate/%	Strata Failure Rate/%
Full caving mining	5394	6000	0.9	0	92	8	34
Full filling	1101	2600	0.18	92	92	8	5
Wide strip mining	357	500	0.06	0	46	46	4
Mixed filling mining	1289	2600	0.21	46	92	8	6
Large mining width—small reserved width	1122	1600	0.19	0	69	31	10
Partial filling mining based on main key strata	1983	3400	0.33	31	92	8	12

## Data Availability

The data supporting reported results can be found by contacting the corresponding author (g_j_zhang@cumt.edu.cn).
